# A Network Pharmacology Study and In Vitro Evaluation of the Bioactive Compounds of *Kadsura coccinea* Leaf Extract for the Treatment of Type 2 Diabetes Mellitus

**DOI:** 10.3390/molecules30051157

**Published:** 2025-03-04

**Authors:** Ying Wang, Shuizhu Cai, Wenzhao Wen, Yanhui Tan, Wenwen Wang, Jing Xu, Ping Xiong

**Affiliations:** Department of Pharmaceutical Engineering, South China Agricultural University, Guangzhou 510642, China

**Keywords:** *Kadsura coccinea*, antidiabetic, network pharmacology, molecular docking

## Abstract

*Kadsura coccinea* is a traditional Chinese medicine whose roots have long been used to treat various ailments, but little is known about the efficacy of its leaves. In this study, the antidiabetic activity of *K. coccinea* leaf extract (KCLE) was determined, the main components of KCLE were identified using UPLC-TOF-MS, and network pharmacology and molecular docking were integrated to elucidate the antidiabetic mechanism of KCLE. The results showed that KCLE effectively increased the glucose consumption of IR-HepG2 cells through pyruvate kinase (PK) and hexokinase (HK), promoted glycogen synthesis, and inhibited α-glucosidase and α-amylase activities. KCLE also improves diabetes by regulating AKT1, TNF, EGFR, and GSK3β. These targets (especially AKT1 and TNF) have a high binding affinity with the main active ingredients of KCLE (rutin, luteolin, demethylwedelolactone, maritimetin, and polydatin). Pathway enrichment analysis showed that the antidiabetic effect of KCLE was closely related to the PI3K-Akt signaling pathway, MAPK signaling pathway, AGE-RAGE signaling pathway, and FoxO signaling pathway. These findings provide a theoretical basis for promoting the pharmacodynamic development of *K. coccinea* and its application in treating diabetes.

## 1. Introduction

Diabetes is a global epidemic that primarily consists of type 1 and type 2 diabetes mellitus (T2DM) [1]. Type 1 diabetes is caused by an autoimmune disorder that causes immune cells to attack insulin-producing β cells of the pancreas, mainly in children and adolescents, while T2DM is caused by poor lifestyle, environmental and genetic factors [2,3]. Diabetes is distinguished by chronic hyperglycemia with disturbances in macromolecule metabolism due to impairments in insulin secretion, insulin action, or both. Diabetes causes long-term damage, dysfunction, and failure of various organ systems (heart, blood vessels, eyes, kidneys, and nerves), leading to disability and premature death [4]. As of 2022, there were about 537 million people with diabetes worldwide, of which T2DM accounts for more than 90% [5]. By 2030, diabetes will be the seventh leading cause of death, according to the World Health Organization [6]. Although synthetic oral hypoglycemic agents and insulin injections are common methods of managing diabetes, they do not seem to be able to completely prevent the development of diabetes complications and sometimes their side effects may exacerbate the condition [7]. Therefore, the search for alternative antidiabetic therapies has become an important research motivation.

Many plants have been considered essential sources of potent antidiabetic drugs for centuries. Nowadays, medicinal plants are suggested for the treatment of diseases including diabetes [8]. Because these plants contain various active ingredients such as flavonoids, terpenoids, saponins, carotenoids, alkaloids, and glycosides, they have antidiabetic activity [9]. In fact, many medicinal plants have been proved to treat diabetes and its complications (namely aloe vera, banana, bitter gourd, caper, cinnamon, cocoa, coffee, fenugreek, garlic, guava, teaspoon ivy, nettles, sage, soy, green and black teas, turmeric, walnuts, and mate) [10]. It can be seen that medicinal plants are an important source of new antidiabetic drugs.

*Kadsura coccinea* is a perennial evergreen climbing woody vine belonging to the family Schisandraceae. Studies on its chemical composition and bioactivity have mainly focused on rhizomes, whose main chemical constituents are lignans, triterpenes, sesquiterpenes, steroids and amino acids [11,12,13]. The rhizomes of *K. coccinea* have high medicinal value and can be used to treat gastric and duodenal ulcers, acute gastroenteritis, rheumatoid arthritis, traumatic injuries, dysmenorrhea and many other diseases [14,15]. Moreover, it has been reported that the fruit extract of *K. coccinea* possesses antidiabetic potential due to its ability to inhibit the activities of α-glucosidase and α-amylase. Its congener, *K. heteroclita*, exhibits similar efficacy, which is attributed to their rich flavonoid content [12]. *K. longipedunculata* has been reported to have an extract, quercetin-3-*O*-rhamnoside, that shows inhibitory activity against α-amylase [16] and its extract can reduce blood glucose levels in mice and ameliorate diabetic nephropathy [17]. These findings underscore the potential of the extracts of *Kadsura* spp. in treating diabetes. However, to date, there have been no reports on the leaf constituents of *K. coccinea* and their application in disease treatment. Flavonoids have been confirmed to have a significant ameliorative effect on diabetes [18]. Current research indicates that the leaves of *K. coccinea* contain a substantial amount of flavonoids, with a higher content than other parts [19], but detailed compositional information is lacking. Therefore, this study aims to identify the main constituents of *K. coccinea* leaves and evaluate their antidiabetic potential.

With the rapid development of bioinformatics, network pharmacology is becoming an effective method to quickly screen active compounds and study potential pharmacological mechanisms. In traditional Chinese medicine research, it is widely used because its completeness and systematicity, which are consistent with traditional Chinese medicine prescription principles [20,21]. Specifically, network pharmacology highlights a paradigm shift from the current “one target, one drug” strategy to a novel version of the “network target, multi-component” strategy [22]. Moreover, with the characteristics of integrity, systematization and high efficiency, it uses bioinformatics, molecular biology and databases to systematically study the relationship between “drug-target-pathway-disease”, and has been widely used in drug research [23,24].

**Figure 1:**
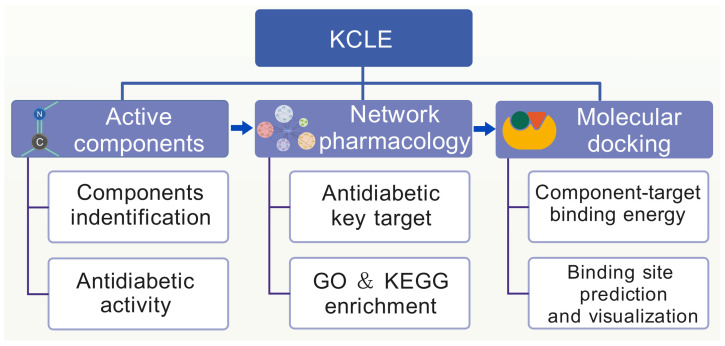
The flowchart of network pharmacology-based strategy for deciphering the mechanisms of KCLE acting on T2DM.

In this study, we used water to extract the active components of *K. coccinea* leaves to obtain *K. coccinea* leaf extract (KCLE). Subsequently, UPLC-Q-TOF/MS was used to determine the chemical composition of KCLE and network pharmacology was used to systematically analyze the active components, potential targets and pathways of the KCLE in the treatment of T2DM. Molecular docking was used to predict the interaction between important compounds and predicted targets. Our results reveal the potential mechanism of the KCLE in treating T2DM and could help to promote the development of new drugs for diabetes. The study workflow is shown in Figure 1.

## 2. Results

### 2.1. Identification of the Chemical Constituents of KCLE

**Figure 2:**
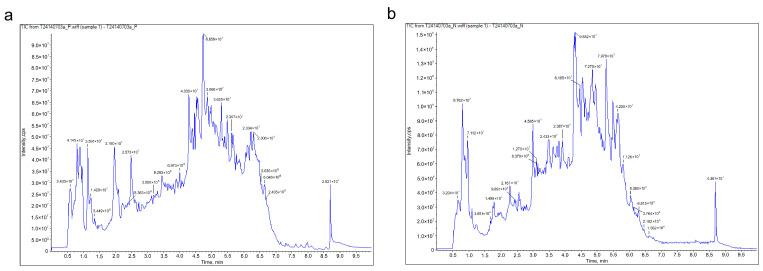
(**a**) The total ion chromatogram (TIC) of KCLE obtained in a positive ion mode. (**b**) The total ion chromatogram (TIC) of KCLE obtained in negative ion mode.

The phytochemical profile of KCLE was characterized by using the UPLC-Q-TOF/MS technique. The total ion chromatogram (TIC) of KCLE is shown in Figure 2. We identified 28 compounds with the largest peak areas, the details of which are listed in Table 1. These compounds included nine flavonoids, five organic acids, four amino acids and their derivatives, two phenolic acids, one vitamin, one lactone, one benzene and its derivative, one heterocyclic compound, one amine compound, one ketone compound, one chromone compound, and one coumarin. Among the top 10 compounds, 5 were flavonoids, with their peak area contribution exceeding 50%, suggesting that flavonoids may be the main active substances responsible for KCLE’s hypoglycemic effects. The root is currently the most intensively studied part of *K. coccinea*. Intriguingly, our findings reveal a divergence in the principal constituents between the leaves and roots. The roots of *K. coccinea* are predominantly abundant in lignans and terpenoids, existing in a remarkable variety and abundance [25], a characteristic that distinguishes them from the leaves. Furthermore, studies have shown that the flavonoid content in the leaves of *K. coccinea* is higher than that in other parts [19]. This further supports our findings that flavonoids are the main components of *K. coccinea* leaves.

### 2.2. The Antidiabetic Potential of KCLE

**Figure 3:**
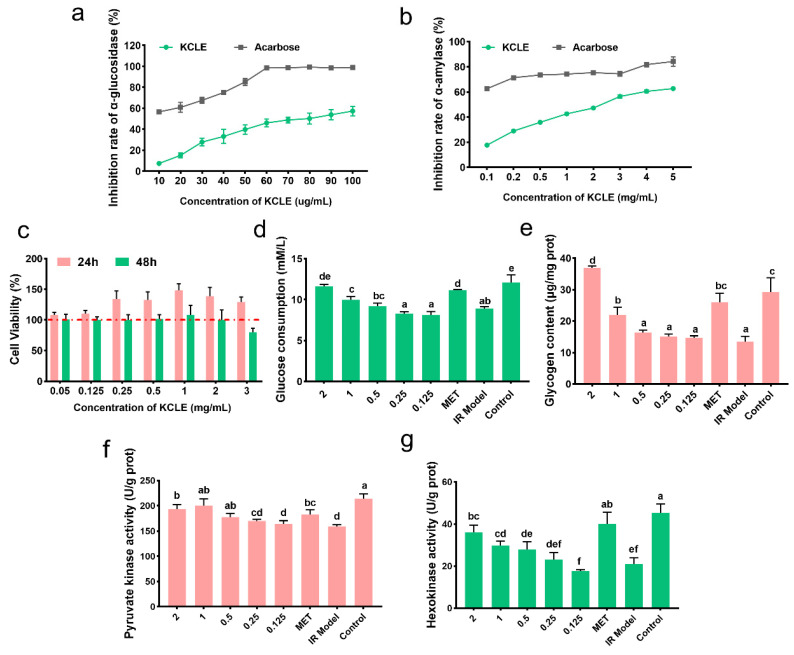
The antidiabetic activity of KCLE. (**a**) Inhibitory effect of KCLE on α-glucosidase. (**b**) Inhibitory effect of KCLE on α- amylase. (**c**) Cell viability of HepG2 cells cultured in different concentrations of KCLE from 0 to 3 mg/mL for 24 h and 48 h. (**d**) Glucose consumption of IR-HepG2 cells incubated with or without KCLE for 24 h; different letters represent significant differences (*p* < 0.05). (**e**) Glycogen content of IR-HepG2 cells incubated with or without KCLE for 24 h; different letters represent significant differences (*p* < 0.05). (**f**) PK activity of IR-HepG2 cells incubated with or without KCLE for 24 h; different letters represent significant differences. (**g**) HK activity of IR-HepG2 cells incubated with or without KCLE for 24 h; different letters represent significant differences.

α-glucosidase and α-amylase can promote the intestinal absorption of sugar. Inhibiting the activities of these two enzymes can effectively lower postprandial blood glucose levels. First, we determined the inhibitory effects of KCLE on α-glucosidase and α-amylase. The results are shown in Figure 3a,b. The use of KCLE effectively inhibited the activities of these two enzymes, especially showing a better inhibitory effect on α-amylase. Secondly, we examined the biosafety and antidiabetic activity of KCLE at the cellular level. As shown in Figure 3c, KCLE had no cytotoxicity to HepG2 cells within the range up to 2 mg/mL and HepG2 cells cultured for 48 h showed obvious cytotoxicity when KCLE concentration reached 3 mg/mL. This suggests that concentrations of KCLE below 2 mg/mL are safe for HepG2. Therefore, the effect of KCLE on cell glucose metabolism was detected using a concentration no higher than 2 mg/mL. Compared with the IR model group, as the concentration of KCLE increased, the consumption of glucose in the culture medium by IR-HepG2 cells also increased (Figure 3d), and KCLE promoted the synthesis of glycogen in IR-HepG2 cells (Figure 3e). This might be attributed to the fact that KCLE simultaneously enhanced the activities of HK and PK (Figure 3f,g), thereby increasing the metabolic rate of glucose in cells. The above results indicate that KCLE has the potential to improve diabetes.

### 2.3. The T2DM-Related Targets of KCLE Constituents

The Lipinski principle is primarily used in the drug design and screening process to help researchers quickly assess whether a compound has the potential to become an oral drug. Compounds that conform to Lipinski’s rule generally have better absorption and metabolic properties [26]. According to Lipinski’s principle, 14 main active components were selected from 28 compounds identified in KCLE. According to previous studies reported that rutin has good biological activity [27]. Therefore, a total of 15 components were identified as potential active ingredients for improving T2DM. Detailed information is shown in Table 2.

**Figure 4:**
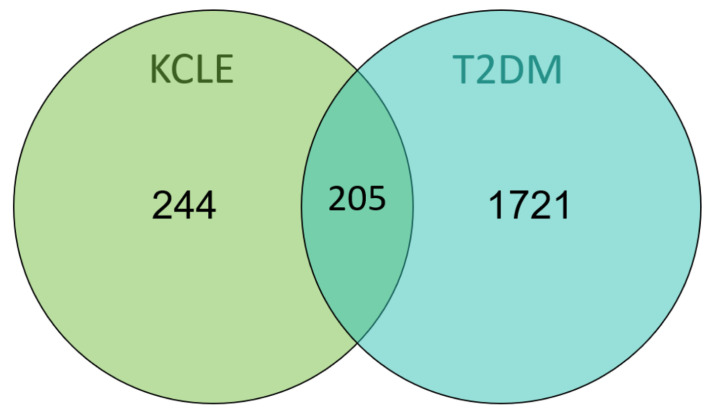
Venn diagram of KCLE and T2DM intersection targets.

After inputting the 15 compounds into SwissTargetPrediction and PharmMapper databases and deleting the duplicate targets, 499 potential targets of KCLE were obtained. By searching the OMIM and TTD databases, a total of 1926 targets related to T2DM were obtained. Then 1926 diabetes-related targets and 499 targets of KCLE constituents were intersected and 205 intersection targets were finally obtained (Figure 4).

### 2.4. Protein-Protein Interaction (PPI) Network Construction 

**Figure 5:**
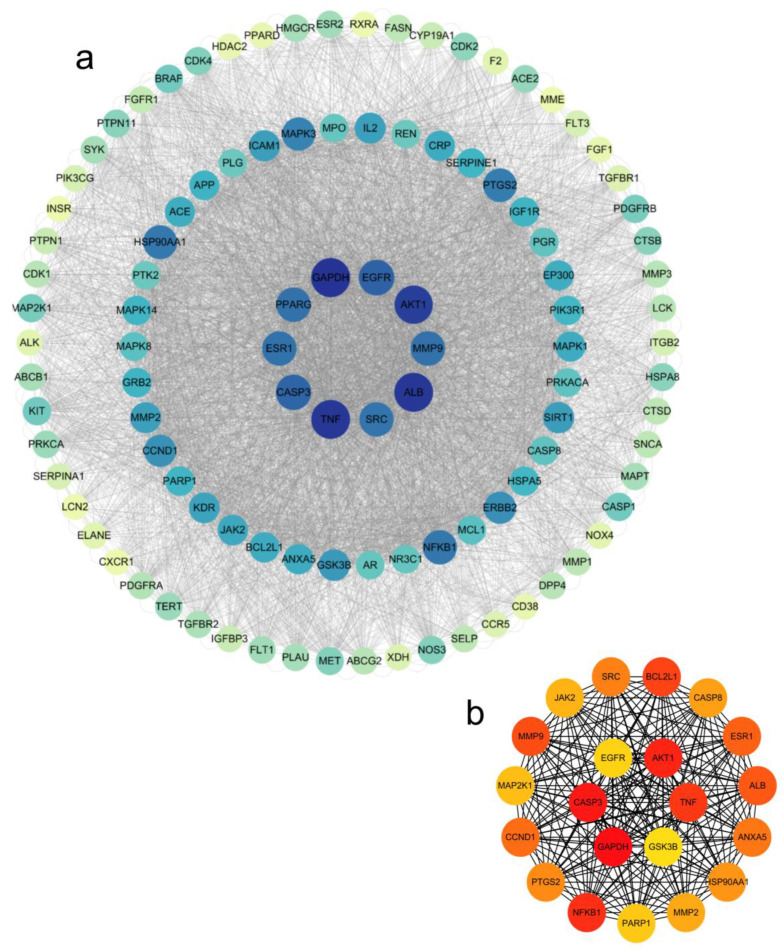
Network pharmacology analysis results. (**a**) PPI network of the common target genes. Diamonds represent proteins, the colors (from blue to green to yellow) indicate the degree of binding between proteins, and the lines represent protein-protein interactions. (**b**) The core target in the PPI network; the color depth represents its interaction strength.

The 205 intersecting targets were input into the STRING database to obtain PPI interaction information. The targets were selected according to the degree value ≥ median and then Cytoscape was used to analyze the network topological characteristics of the PPI network and visualize the results, as shown in Figure 5a, with a total of 106 nodes and 4956 edges. Using the CytoHubba plugin in Cytoscape, the MMC algorithm was used to analyze and identify 21 core targets (AKT1, TNF, GSK3B, GAPDH, CASP3, EGFR, BCL2L1, CASP8, ESR1, ALB, ANXA5, HSP90AA1, MMP2, PARP1, NFKB1, PTGS2, CCND1, MAP2K1, MMP9, JAK2, and SRC) as shown in Figure 5b, indicating that KCLE may exert hypoglycemic effects through multiple targets.

### 2.5. Component-Pathway-Target Network Construction

**Figure 6:**
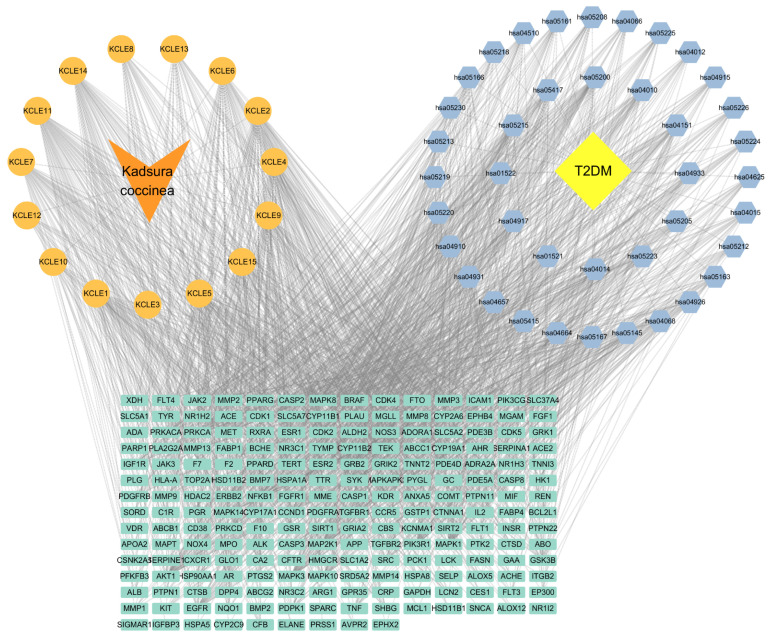
Compound-Gene-Pathway network. Green rectangles represent hub genes, orange circles represent active compounds, and blue diamonds indicate pathways associated with the core targets.

We selected 20 active ingredients, 111 potential target sites and the first 40 pathways to construct a component-pathway-target network diagram, as shown in the Figure 6. The specific topology parameters of the network are shown in Table 3. Betweenness Centrality is a metric used to measure the importance of nodes in a network, which reflects the frequency of occurrence of nodes along the shortest path in the network [28]; Degree represents the number of edges directly connected to a node [29]. The greater the values of the two parameters, the more important the target is considered to be. Through an analysis of the degree values of target sites, the top 10 target sites ranked were: AKT1, MAPK1, MAPK3, PIK3R1, EGFR, MAP2K1, MAPK8, MAPK10, TNF, and GSK3B. According to degree analysis, KCLE2, KCLE11, KCLE6, KCLE14, KCLE3, KCLE7, KCLE5, KCLE9, KCLE4, and KCLE1 are 10 important compounds with the potential to play a role in the treatment of diabetes.

### 2.6. GO Enrichment Analysis

**Figure 7:**
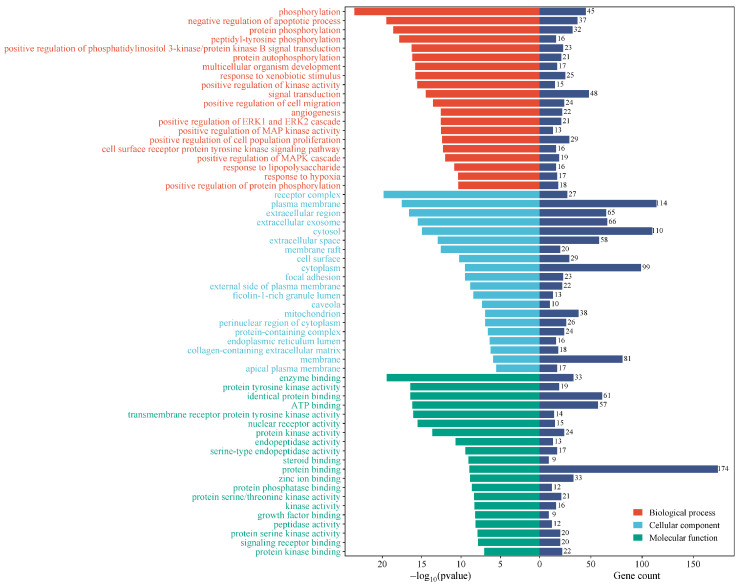
Visualization of term enrichment. Gene count represents the number of targets corresponding to all targets in that term.

The KCLE therapeutic targets related to T2DM were subjected to GO analysis, and a total of 462 GO terms were obtained (*p* < 0.01) including 323 terms for biological process, 54 terms for cellular component, and 85 terms for molecular function. The top 20 terms are illustrated in Figure 7. The biological process mainly involved phosphorylation, negative regulation of the apoptosis process, protein phosphorylation, peptide tyrosine phosphorylation, positive regulation of phosphatidylinositol 3-kinase/protein kinase B signal transduction, etc. The cellular components mainly involved receptor complexes, cytoplasmic membranes, extracellular regions, extracellular exosomes, cytosol, etc. Molecular functions mainly involve protein tyrosine kinase activity, ATP binding, transmembrane receptor protein tyrosine kinase activity, nuclear receptor activity, protein kinase activity, etc.

### 2.7. KEGG Pathway Analysis

**Figure 8:**
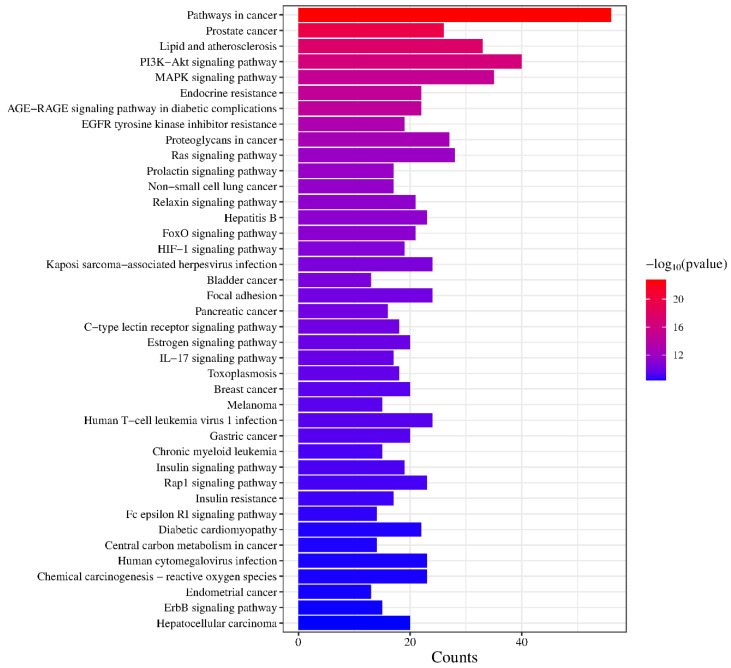
Visualization of KEGG pathway enrichment.

KEGG pathway enrichment analysis yielded 150 pathways (*p* < 0.01) and the top 40 pathways were selected for visualization, as shown in the Figure 8. Among the top 40 pathways, the overlapping genes were densely mapped to Pathways in cancer, Prostate cancer, Lipid and atherosclerosis, PI3K-Akt signaling pathway, MAPK signaling pathway, Endocrine resistance, AGE-RAGE signaling pathway in diabetic complications, EGFR tyrosine kinase inhibitor resistance, Proteoglycans in cancer, Ras signaling pathway, Prolactin signaling pathway, Non-small cell lung cancer, Relaxin signaling pathway, Hepatitis B, FoxO signaling pathway, etc. In addition, the predicted pathways (*p* < 0.01) included the Insulin signaling pathway, Insulin resistance, Estrogen signaling pathway, Diabetic cardiomyopathy, C-type lectin receptor signaling pathway, and IL-17 signaling pathway, suggesting that the active components of KCLE may exert hypoglycemic effects by regulating immune, insulin, and inflammation pathways.

### 2.8. Molecular Docking of Core Components with Key Targets

**Figure 9:**
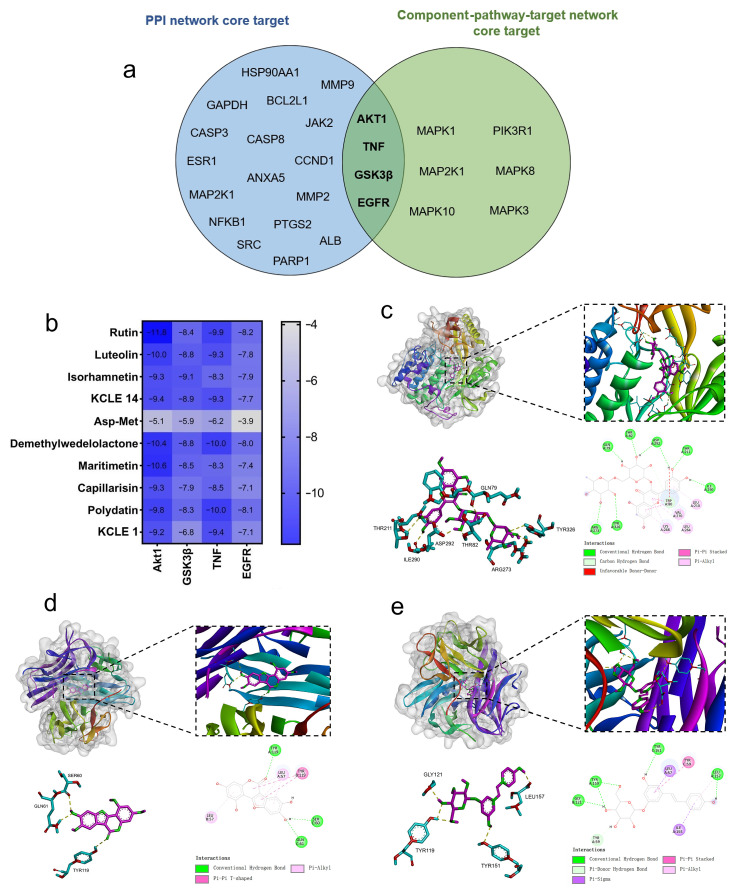
Molecular docking results of the important compounds and the corresponding proteins of the gene targets. (**a**) Venn diagram of PPI network core targets and component-pathway-target network core targets. (**b**) The binding energy of the target protein and the active ingredient, measured in kcal/mol, indicates a stable binding when the binding energy is less than −5.0 kcal/mol. (**c**) Visualization of the docking results of AKT1 and rutin. (**d**) Visualization of the docking results of TNF and demethylwedelolactone. (**e**) Visualization of the docking results of TNF and polydatin.

The ten important active constituents were chosen for molecular docking verification with the key targets TNF, AKT1, EGFR, and GSK3β (the core intersection target of the PPI network and Component-pathway-target network, Figure 9a), which are considered potential targets in the treatment of diabetes. The binding energy (Vina score) calculated by AutoDock Vina was used to estimate the bonding activity between the docking molecules, with a smaller Vina score indicating stronger bonding activity as well as a higher affinity and more stable structure between the ligand and receptor. The docking scores between the key target proteins and small molecule active components are shown in Figure 9b. All active components except Asp-Met showed good docking and high affinity with key target proteins (Vina score < −5.0 kcal/mol). Among them, Akt1 and TNF have the lowest binding energies with all the active components, which suggests that Akt1 and TNF are the main targets of KCLE to reduce blood glucose. Playing the role of the main composition are rutin, luteolin, demethylwedelolactone, maritimetin, and polydatin.

We visualized the active component-target (Akt1 and TNF) interactions with the lowest free binding energy score and their binding modes using PyMol and Discovery Studio (Figure 9c–e). It can be observed that eight hydrogen bonds are formed between the rutin molecule and the residues THR211, ILE290, ASP292, THR82, ARG273, TYR326, and GLN79 in the active pocket of Akt1 protein. Meanwhile, three hydrogen bonds are formed between the demethylwedelolactone molecule and the residues SER60, GLN61, and TYR119 in TNF. Additionally, four hydrogen bonds are formed between the polydatin molecule and the residues GLY121, LEU157, TYR119, and TYR151 in TNF. By hydrogen bonding, hydrophobic small molecules and active cavities of target proteins form stable complexes.

## 3. Discussion

*K. coccinea* is a traditional folk medicinal plant with various medicinal activities in its rhizome. However, little is known about the main active components in its leaves and their effects. Here, we identified the main components of KCLE and reported for the first time its potential application and mechanism of action in antidiabetes. In recent years, benefiting from the rapid development of bioinformatics technology, network pharmacology analysis and molecular docking technology have been used to screen active ingredients and potential targets, evaluate the degree of complex-target binding, and explain the pharmacological effects of traditional Chinese medicine [30]. The application of these tools can accelerate the elucidation of the mechanism of action of medicinal plants and the development of new drugs.

We identified 28 major compounds in KCLE by UPLC-TOF-MS (9 flavonoids, 5 organic acids, 4 amino acids and their derivatives, 2 phenolic acids, 1 vitamin, 1 lactone, 1 benzene and its derivative, 1 heterocyclic compound, 1 amine compound, 1 ketone compound, 1 chromone compound, and 1 coumarin), and then selected 15 compounds with potential activity for network pharmacology study based on Lipinski’s principle of drug-like properties, including rutin (flavonoid), demethylwedelolacton (coumarin), maritimetin (flavonoid), isorhamnetin (flavonoid), polydatin (organic acids), 1-{[3-(3,4-Dihydroxyphenyl)acryloyl]oxy}-3,5-dihydroxycyclohexane-1-carboxylic acid (organic acids), Asp-Met (amino acids and their derivatives), 3-*p*-Coumaroylquinic acid (phenolic acid), capillarisin (chromone), protocatechuic acid (phenolic acid), luteolin (flavonoid), perillaldehyde (heterocyclic compound), menadione (vitamin), glucovanillin (benzene and its derivative), and 3,3′,5,5′,7-pentahydroxyflavane (ketone compound). Among these active ingredients, rutin, isorhamnetin, maritimetin, and luteolin are flavonoids that have been reported to have a hypoglycemic effect. An increasing amount of evidence indicates that flavonoids derived from vegetables and medicinal plants can be beneficial for diabetes by improving glucose control, lipid levels, and antioxidant status [18]. Previous studies have shown that rutin can improve diabetes by inhibiting the activities of α-glucosidase and α-amylase [31,32], activating the synthesis of transporter GLUT-4 [33], increasing the expression of PPARγ [28], reducing hepatic G6Pase [34], and increasing hexokinase activity [33]. Isorhamnetin can promote carbohydrate metabolism through digestion and intestinal absorption, improve glucose uptake in liver and muscle [35], and alleviate the negative effects of diabetes by promoting GLUT4 translocation and regulating intestinal flora [36,37]. Luteolin ameliorated hyperglycemia and improved hypoinsulinemia, β-cell dysfunction, and renal impairment in HFD-STZ-induced diabetic rats by attenuating inflammation and dysregulated cytokine secretion through modulation of PPAR-γ, TNF-α, IL-6, and NF-kB expression and down-regulation of SREBP-1c [38]. Maritimetin has been studied less for its antidiabetic properties, but there have been reports suggesting that it may improve insulin resistance [39]. In summary, flavonoids are a class of important natural active substances that can improve diabetes. Additionally, polydatin [40,41], dimethylwedelolactone [42], capillarisin [43], protocatechuic acid [44], perillaldehyde [45], and menadione [46] have also been reported to have beneficial effects on diabetes or diabetes complications, which has not yet been seen with 1-{[3-(3,4-Dihydroxyphenyl)acryloyl]oxy}-3,5-dihydroxycyclohexane-1-carboxylic acid, 3-*p*-Coumaroylquinic acid, glucovanillin, and 3,3′,5,5′,7-pentahydroxyflavane in relation to the improvement of diabetes mellitus. This may require further experimental confirmation.

According to the results of network pharmacological analysis, the above 15 active ingredients exert antidiabetic effects through a multi-target multi-pathway. Analysis of the compose-gene-pathway network showed that rutin, luteolin, demethylwedelolactone, maritimetin, and polydatin were the main active components of KCLE. They exert antidiabetic effects through TNF, AKT1, EGFR, GSK3β, and other targets. By simulating the affinity of the active ingredient to the target through molecular docking, we further determined that Akt1and TNF are the main targets of KCLE for hypoglycemia due to its lower binding energy with multiple active ingredients. These two targets are strongly associated with diabetes. AKT1 is one of three AKT kinases that can control glucose absorption into fat cells and muscles by increasing GLUT4 glucose transporter translocation. By suppressing the expression of glucose 6-phosphatase and phosphoenolpyruvate carboxykinase, Akt also represses liver gluconeogenesis [47]. TNF is one of the most important pro-inflammatory mediators and plays a crucial role in the development of IR and pathogenesis of T2DM [48,49]. It can promote JNK phosphorylation, which inhibits IRS-2/PI3K/Akt/GSK-3β pathway, resulting in impaired glucose uptake and glycogen synthesis [50,51,52]. In addition, in previous antidiabetes studies, rutin, luteolin, maritimetin, and polydatin have also shown high affinity with other diabetes-related targets (Table 4). Rutin shows high affinity with AKT1 and EGFR [53], and also with α-glucosidase and α-amylase [54]; luteolin with AKT1, VEGFA, NOS3, PPARG, MMP9, VCAM1, which has a high binding activity [55]; maritimetin has a good affinity with the diabetes drug target 5NN8 [56]; and polydatin also showed high affinity with PCSK9 and STING [41,57]. These findings reveal additional potential and value of these core components in treating diabetes. One limitation of the present study is that biotransformation was not considered. Once the compound enters the body, it may be transformed in the gastrointestinal environment [58]. For instance, the primary acidic substance in gastric acid is Hcl [59], which promotes the hydrolysis of glycosides of polydatin to produce resveratrol [60]. From this point, it is evident that there are limitations to utilizing molecular docking technology to investigate the binding affinity of compounds with target proteins.

GO functional enrichment analysis, KCLE antidiabetics mainly through protein phosphorylation, peptide tyrosine phosphorylation, positive regulation of phosphatidylinositol 3-kinase/protein kinase B signal transduction, and other biological processes affect diabetes. KEGG functional enrichment analysis showed that the PI3K-Akt signaling pathway [61], MAPK signaling pathway [62], AGE-RAGE signaling pathway [63], which are closely related to diabetes mellitus, and the FoxO signaling pathway are significantly enriched [64], and these pathways have been shown by many studies to play an important role in antidiabetes.

Cell culture experiments also showed that KCLE has good biosafety and can effectively improve glucose consumption of IR-HepG2 cells by increasing PK and HK activities. In addition, KCLE can also inhibit α-glucosidase and α-amylase and reduce the absorption of sugar by the intestine; it may be that rutin played a major role [31,32,53]. Our findings therefore further confirm that the main active ingredient of KCLE may play an important role in the treatment of diabetes. Subsequent research will mainly focus on experiments with diabetic animal models to further investigate the antidiabetic effects and mechanisms of KCLE.

## 4. Materials and Methods

### 4.1. Experimental Material Sources

The HepG2 cell was obtained from Dr. Hong (South China Agricultural University). α-glucosidase and α-amylase were purchased from Shanghai yuanye Bio-Technology Co., Ltd. (Shanghai, China); PBS and DMEM were purchased from Yeasen Biotechnology Co., Ltd. (Shanghai, China); insulin was purchased from Novo Nordisk China Pharmaceuticals Co., Ltd. (Tianjin, China); all other reagents were purchased from Shanghai Macklin Biochemical Technology Co., Ltd. (Shanghai, China); the purity of reagents used in UPLC-Q-TOF/MS is all chromatographic grade.

### 4.2. KCLE Chemical Composition Detection

*K. coccinea* was sourced from the *K. coccinea* cultivation base in Lishong Town, Hezhou City, Guangxi Zhuang Autonomous Region, China. It was store at 4 °C. After the washed leaves were dried at room temperature, they are crushed using a grinder. The *K. coccinea* leaf powder was extracted by water bath heating condensation reflux (KCL: water= 1:30). Reflux extraction was performed three times at 100 °C, each time for 75 min. The water extract was mixed and concentrated with a rotary evaporator. The concentrated liquid was combined and freeze-dried for 48 h under vacuum to obtain the lyophilized powder of *K. coccinea* leaf extract.

Weigh out 50 mg of the KCLE lyophilized powder and add 1200 μL of a precooled (−20 °C) 70% methanol. The internal standard extraction solution was prepared by dissolving 1 mg of the standard (2-Chloro-L-phenylalanine) in 1 mL of 70% methanol to prepare a 1000 μg/mL standard mother solution. The 1000 μg/mL mother solution was further diluted with 70% methanol to prepare a 250 μg/mL internal standard solution. A vortex mixer was used to vortex the samples every 30 min for 30 s for a total of six times. Then, centrifuge at 12,000 rpm for 3 min, take the supernatant, filter the sample using a 0.22 μm filter, and store it in a sample vial for UPLC-MS/MS analysis.

UPLC was performed on a Shimadzu LC-30A Liquid Chromatograph system (Shimadzu Corp, Kyoto, Japan). Chromatographic separations were performed using a Waters ACQUITY Premier HSS T3 Column (100 mm × 2.1 mm, 1.8 μm) (Waters Corp, Milford, MA, USA). The column temperatures was maintained at 40 °C. The mobile phase was composed of solvents A (formic acid/water = 0.1/100, *v*/*v*) and B (acetonitrile/formic acid = 0.1/100, *v*/*v*). The gradient program was optimized as follows: 0–2 min, 5–20% B; 2–5 min, 20–60% B; 5–6 min, 60–99% B; 6–7.5 min, 99% B; 7.5–7.6 min, 99–5% B; 7.6–10 min, 5%. The flow rate was set at 0.4 mL/min. The injection volume of the reference compounds and samples was 4 μL.

The data acquisition was operated using the information-dependent acquisition (IDA) mode using Analyst TF 1.7.1 Software (Sciex, Concord, ON, Canada). The source parameters were set as follows: ion source gas 1 (GAS1), 50 psi; ion source gas 2 (GAS2), 50 psi; curtain gas (CUR), 25 psi; temperature (TEM), 550 °C; declustering potential (DP), 60 V or −60 V in positive or negative modes, respectively; and ion spray voltagefloating (ISVF), 5000 V or −4000 V in positive or negative modes, respectively. The TOF MS scan parameters were set as follows: mass range, 50–1000 Da; accumulation time, 200 ms; and dynamic background subtract, on. The product ion scan parameters were set as follows: mass range, 25–1000 Da; accumulation time, 40 ms; collision energy, 30 or −30 V in positive or negative modes, respectively; collision energy spread, 15; resolution, UNIT; charge state, 1 to 1; intensity, 100 cps; exclude isotopes within 4 Da; mass tolerance, 50 ppm; maximum number of candidate ions to monitor per cycle, 18.

### 4.3. In Vitro Validation

#### 4.3.1. The Inhibitory Effect of α-Glucosidase

With 0.1 M of phosphate buffer solution (PBS) mixture, KCLE solutions of different concentrations (10, 20, 30, 40, 50, 60, 70, 80, 90, 100 μg/mL), α-glycosidase enzyme solution (0.2 U/mL), and pNPG solution (2.5 mM) were used. Next, 0.1 M phosphoric acid buffer solution 120 μL, 0.2 U/mL α-glucosidase solution 20 μL, and KCLE solution 20 μL were added into 96-well plates and reacted in a 37 °C constant temperature incubator for 10 min. Then 20 μL pNPG solution was added and continued to react in a 37 °C constant temperature incubator for 20 min. Finally, 80 μL Na_2_CO_3_ solution of 0.2 mol/L was added to stop the reaction. Finally, the absorbance was measured at a wavelength of 405 nm and acarbose was used for the positive control. Every experiment was conducted with six replicates.

#### 4.3.2. The Inhibitory Effect α-Amylase

Different concentrations of KCLE solution (0.1, 0.2, 0.5, 1, 2, 3, 4, 5 mg/mL), α-amylase solution (50 U/mL), and soluble starch solution (1%) were prepared with 0.1 mol/L PBS. Next, 30 µL of KCLE solution and α-amylase solution was measured in a centrifuge tube, reacted at 37 °C for 10 min, then added to the same volume of soluble starch solution, boiled in a water bath for 15 min, rapidly cooled, and 50 µL DNS added to it, then it was heated at 95 °C for 5 min, and 500 µL of distilled water added after cooling. The absorbance value of the solution at 540 nm was detected with acarbose as the positive control. Every experiment was conducted with six replicates.

#### 4.3.3. Effect of KCLE on HepG2 and IR-HepG2

Next, 200 µL HepG2 cells were added to the 96-well plate (5 × 10^4^ cells/well). After 12 h of pre-adhesion at 37 °C, 100 µL of KCLE with different concentrations (0.05, 0.125, 0.25, 0.5, 1, 2, 3 mg/mL) was added and incubated in an incubator at 37 °C for 24 h and 48 h. After the incubation, the medium was removed. Subsequently, 200 µL MTT (0.5 mg/mL) was added to each well for 4 h. After that, the supernatant was removed and 150 µL of DMSO was added to each well and shaken for 10 min. The treatment without KCLE was used as a control group to calculate cell survival. Finally, the absorption value was measured at 490 nm. Every experiment was conducted with six replicates.

The insulin resistance model of HepG2 cells was established. HepG2 cells were cultured in a cell incubator (37 °C) for 24 h and then starved in serum-free medium for 24 h. Then high glucose DMEM containing recombinant human insulin (0.01 mM) was used to replace serum-free medium at 37 °C for 48 h to establish IR model, before 2 mL IR-HepG2 cells (5 × 10^5^ cells/well) were added to the 6-well plate. After 24 h of pre-adhesion at 37 °C, different concentrations of KCLE (0.125, 0.25, 0.5, 1, 2 mg/mL) were used to replace the original medium for further culture at 37 °C for 24 h. Subsequently, the glucose content, cellular glycogen content, and cellular hexokinase (HK) and pyruvate kinase (PK) activities in the medium were measured using a kit. IR-HepG2 supplemented with metformin was used as the positive control group, IR-HepG2 supplemented without KCLE and metformin was used as the negative control. In addition, normal HepG2 cells were used as the control group to compare the therapeutic effects of KCLE treatment and metformin treatment on IR-HepG2 cells, following the kit manufacturer’s instructions. The above kits were purchased from Nanjing Jiancheng Bioengineering Research Institute Co., Ltd (Nanjing, China). Every experiment was conducted with six replicates.

### 4.4. Active Component Screening and Target Prediction of KCLE

The main components of KCLE were obtained from Section 4.1. The PubChem database (https://pubchem.ncbi.nlm.nih.gov/, accessed on 1 November 2024) was used to obtain the chemical structure formula and SMILES formula of the chemical composition of KCLE [65]. The chemical composition structure formula or SMILES formula was imported into the Swiss ADME database (http://www.swissadme.ch/, accessed on 1 November 2024) [66], and the chemical composition was screened according to the Lipinski insecticide principle. Then the Swiss Target Prediction database (http://www.swisstargetprediction.ch/, accessed on 2 November 2024) and PharmMapper database (http://lilab-ecust.cn/pharmmapper/index.html, accessed on 2 November 2024) were used to predict the potential target genes of the selected components [67,68]. The corresponding target genes were searched through the Uniprot database (https://www.uniprot.org/, accessed on 3 November 2024) [69], and the potential target genes of KCLE chemical components were obtained by merging and removing duplication.

### 4.5. Construction of Drug Component-Target Network

The collected active components and potential targets of KCLE were imported into Cytoscape (v 3.9.1) to construct the drug component-target network. The Network Analyzer tool was used to calculate the topological parameters and then the component-target network was analyzed according to the degree value.

### 4.6. Screening the Targets Related to T2DM

The Human Genome Annotation (GeneCards) database (https://www.genecards.org/, accessed on 5 November 2024) and OMIM database (https://www.omim.org/, accessed on 5 November 2024) were used to obtain the related target genes with “Type 2 Diabetes Mellitus” as the search term [70,71]. The Uniprot database was used to standardize, merge and remove duplication, and the target genes of T2DM were obtained.

### 4.7. Construction of Protein-Protein Interaction (PPI) Network

In order to collect the overlapping targets of drugs and diseases, we imported the potential active ingredient targets and T2DM targets of KCLE into the Venn database (http://www.bioinformatics.com.cn/static/others/jvenn/example.html, accessed on 7 November 2024). The targets in the intersection were imported into the STRING database (https://string-db.org/, accessed on 7 November 2024) to obtain information on the protein interaction network [72]. The screening condition of the organism is set to “Homo sapiens” and the minimum required interaction score is “highest confidence (0.9)”. The protein-protein interaction (PPI) information was input into Cytoscape and the targets whose degree value was greater than the median were selected for visualization and a PPI network constructed. The MCODE and CytoHubba plugins were then used to obtain key modules and screen key targets.

### 4.8. GO Enrichment and KEGG Pathway Analysis

To study the biological function of potential targets in T2DM, the DAVID (https://david.ncifcrf.gov/, accessed on 8 November 2024) database was used to collect GO analysis and KEGG data [73]. GO analysis is used to screen biological processes (BPs), cellular components (CCs), and molecular functions (MFs). KEGG enrichment analysis can find important signaling pathways involved in biological processes. Subsequently, GO and KEGG data were uploaded to the Bioinformatics (http://www.bioinformatics.com.cn/, accessed on 8 November 2024) platform for visual analysis.

### 4.9. Molecular Docking

According to the results of network pharmacology analysis, 2D structures of active compounds were downloaded from the PubChem database and imported into the Chem3D software for energy minimization. Other parameters were set to default values. The crystal structure of the core target protein was downloaded from the PDB database and the target protein information is shown in the table. The crystal structure of the target protein was imported into the PyMol and the water molecules and small molecule ligands were removed. Then, the protein was hydrogenated using AutoDock software (v 1.5.7) and the optimized small molecule ligand was imported. The active center for docking was set using the grid box function in the software and molecular docking analysis was performed using Vina software. Finally, the docking results were visualized using Discovery Studio software.

## 5. Conclusions

KCLE has significant antidiabetic potential and can promote the glucose consumption of IR-HepG2 cells by increasing the PK and HK activities, while inhibiting the activity of α-glucosidase and α-amylase. Moreover, network pharmacology and molecular docking results show that rutin, luteolin, demethylwedelolactone, maritimetin, and polydatin in KCLE are the core active compounds of KCLE in treating diabetes. With AKT1 and TNF as core targets, they exert antidiabetic effects through the PI3K-Akt signaling pathway, MAPK signaling pathway, AGE-RAGE signaling pathway, and FoxO signaling pathway. Our research findings could help develop new antidiabetic dietary supplements or drugs and provide new evidence for the use of KCLE in diabetes treatment.

## Figures and Tables

**Table 1:** Compounds identified in KCLE.

No.	RT (min)	Compound Name	Area	Molecular Formula	Molecular Weight	CAS
1	0.8017	Menadione	4411706	C_11_H_8_O_2_	172.0524	58-27-5
2	0.8884	Isobrucein B	4681534	C_23_H_28_O_11_	480.1632	53663-03-9
3	0.8931	Glucovanillin	3700869	C_14_H_18_O_8_	314.1002	494-08-6
4	2.0185	L-Phenylalanine	12891600	C_9_H_11_NO_2_	165.079	63-91-2
5	2.4974	L-Tryptophan	8704845	C_11_H_12_N_2_O_2_	204.0899	73-22-3
6	2.5601	Protocatechuic acid	5126220	C_7_H_6_O_4_	154.0266	99-50-3
7	2.9397	3-*p*-Coumaroylquinic acid	6461959	C_16_H_18_O_8_	338.1002	1899-30-5
8	3.0082	1-{[3-(3,4-Dihydroxyphenyl)acryloyl]oxy}-3,5-dihydroxycyclohexane-1-carboxylic acid	19031736	C_16_H_18_O_8_	338.1002	153444-59-8
9	4.2931	Quercetin 3-(2-glucosylrhamnoside)	22262972	C_27_H_30_O_16_	610.1534	143016-74-4
10	4.2967	Rutin	16009941	C_27_H_30_O_16_	610.1534	153-18-4
11	4.3096	Vincetoxicoside A	4711617	C_27_H_30_O_16_	610.1534	18016-58-5
12	4.5683	3′-*O*-L-Rhamnopyranosylastragalin	12646128	C_27_H_30_O_15_	594.1585	28447-29-2
13	4.5999	Isorhamnetin 3-*O*-alpha-rhamnopyranosyl-(1-2)-beta-galactopyranoside	9447197	C_28_H_32_O_16_	624.169	107740-46-5
14	4.6362	N-Tris(hydroxymethyl)methyl-2-aminoethanesulfonic acid	5540480	C_6_H_15_NO_6_S	229.062	7365-44-8
15	4.7988	Perillaldehyde	4590032	C_10_H_14_O	150.1045	2111-75-3
16	5.2659	Sodium 5-(diphenylphosphinoyl)pentanoate	3740540	C_17_H_18_NaO_3_P	324.0891	-
17	5.2847	Polydatin	12691776	C_20_H_22_O_8_	390.1315	27208-80-6
18	5.2877	3,3′,5,5′,7-pentahydroxyflavane	4170465	C_15_H_10_O_7_	302.0427	28449-61-8
19	5.4946	Asp-Met	14929153	C_9_H_16_N_2_O_5_S	264.078	-
20	5.5101	Morin hydrate	5030901	C_15_H_12_O_8_	320.0532	654055-01-3
21	5.5163	Demethylwedelolactone	7747091	C_15_H_8_O_7_	300.027	6468-55-9
22	5.6226	Maritimetin	10561988	C_15_H_10_O_6_	286.0477	576-02-3
23	5.6294	Luteolin	4979921	C_15_H_10_O_6_	286.0477	491-70-3
24	5.6894	Capillarisin	5468023	C_16_H_12_O_7_	316.0583	56365-38-9
25	5.7716	Isorhamnetin	8234449	C_16_H_12_O_7_	316.0583	480-19-3
26	6.1267	N-Nitrosodibutylamine	3913966	C_8_H_18_N_2_O	158.1419	924-16-3
27	6.2324	Ala-Ala-Gln-Asn-Leu	4013332	C_21_H_37_N_7_O_8_	515.2704	-
28	8.7134	{5-[(E)-2-(3,5-dihydroxyphenyl)ethenyl]-2-hydroxyphenyl}oxidanesulfonic acid	3948958	C_14_H_12_O_7_S	324.0304	-

**Table 2:** Information on bioactive constituents of KCLE.

ID	Compound Name	Lipinski Rules				Lipinski Violations ≤ 1	Bioavailability Score > 0.1
		MW ≤ 500	MLOGP ≤ 4.15	H-Bond Acceptors ≤ 10	H-Bond Donors ≤ 5		
KCLE1	1-{[3-(3,4-Dihydroxyphenyl)acryloyl]oxy}-3,5-dihydroxycyclohexane-1-carboxylic acid	338.31	−0.27	8	5	0	0.56
KCLE2	Rutin	610.52	−3.89	16	10	3	0.17
KCLE3	Asp-Met	264.30	−0.88	6	4	0	0.11
KCLE4	Polydatin	390.38	−0.36	8	6	1	0.55
KCLE5	Maritimetin	286.24	0.09	6	4	0	0.55
KCLE6	Isorhamnetin	316.26	−0.31	7	4	0	0.55
KCLE7	Demethylwedelolactone	300.22	0.41	7	4	0	0.55
KCLE8	3-*p*-Coumaroylquinic acid	338.31	−0.54	8	5	0	0.56
KCLE9	Capillarisin	316.26	0.37	7	3	0	0.55
KCLE10	Protocatechuic acid	154.12	0.40	4	3	0	0.56
KCLE11	Luteolin	286.24	−0.03	6	4	0	0.55
KCLE12	Perillaldehyde	150.22	2.10	1	0	0	0.55
KCLE13	Menadione	172.18	1.20	2	0	0	0.55
KCLE14	3,3′,5,5′,7-pentahydroxyflavane	302.24	−0.56	7	5	0	0.55
KCLE15	Glucovanillin	314.29	−1.83	8	4	0	0.55

**Table 3:** Topology parameters of important compounds and targets in the network.

Component	Betweenness Centrality	Degree	Target	Betweenness Centrality	Degree
KCLE2	0.131469	89	AKT1	0.033568794	46
KCLE11	0.081337	77	MAPK1	0.022559678	42
KCLE6	0.067662	71	MAPK3	0.017069732	39
KCLE14	0.055473	66	PIK3R1	0.016442178	39
KCLE3	0.10808	64	EGFR	0.029796967	37
KCLE7	0.062448	55	MAP2K1	0.010038906	34
KCLE5	0.050035	54	MAPK8	0.029681602	33
KCLE9	0.035002	52	MAPK10	0.019960132	32
KCLE4	0.044135	50	TNF	0.022131055	28
KCLE1	0.077422	50	GSK3β	0.020604069	28

**Table 4:** High affinity targets of five core components in other studies.

Component	Target	References
Rutin	AKT1, EGFR, α-glucosidase, α-amylase	[53,54]
Luteolin	AKT1, VEGFA, NOS3, PPARG, MMP9, VCAM1	[55]
Maritimetin	5NN8	[56]
Polydatin	PCSK9, STING	[41,57]

## Data Availability

Data are available from the corresponding authors upon reasonable request.
